# Dr. Elton S. Rich

**Published:** 1908-11

**Authors:** 


Dr. Elton S. Rich, of Kennedy, N. Y., was found dead in his office, October 7, 1908, aged 54 years. He graduated from New York University in 1886, and was one of the veteran physicians in Chautauqua County. He practised his profession at Kennedy for some twenty years or more.

